# Clearing the confusion about post-accreditation monitoring, meta-evaluation and meta-accreditation

**DOI:** 10.1186/s12909-024-05214-7

**Published:** 2024-03-07

**Authors:** Roghayeh Gandomkar, Azim Mirzazadeh, Tahereh Changiz

**Affiliations:** 1 Department of Medical Education, School of Medicine, Tehran University of Medical Sciences, Tehran, Iran; 2 Health Profession Education Research Center, Tehran University of Medical Sciences, Tehran, Iran; 3 Department of Internal Medicine, School of Medicine, Tehran University of Medical Sciences, Tehran, Iran; 4 Department of Medical Education, Isfahan University of Medical Sciences, Isfahan, Iran; 5 Medical Education Research Center, Isfahan University of Medical Sciences, Isfahan, Iran; 6 Department of Medical Education, No. 57, Hojjatdust Alley, Naderi St., Keshavarz Blvd, 141663591 Tehran, Iran

**Keywords:** Post-accreditation monitoring, Meta-evaluation, Meta-accreditation

## Abstract

We have recently published the experience of the accreditation body of undergraduate medical education in Iran on developing and validating standards based on the WFME framework (Gandomkar et al., BMC Med Educ 23:379, 2023). Agabagheri et al. extended our work and proposed a blueprint for post-accreditation monitoring based on their experience in developing an official guide in their Matters Arising (Aghabagheri et al., BMC Med Educ). The authors have used post-accreditation monitoring as a process of monitoring and controlling accreditation activities, procedures often referred to as meta-evaluation or meta-accreditation (depending on the objectives of evaluation) in the literature. On the contrary, post-accreditation monitoring alludes to the process of continuous quality improvement of educational programs after accreditation. We would like to make clarifications between post-accreditation monitoring, meta-evaluation and meta-accreditation which have been used interchangeably in their paper. Considering the emerging interests in scholarship and non-scholarship activities and reports in undergraduate medical education accreditation, this clarification provides a better understanding of the roles of these crucial concepts in the accreditation process.

Accreditation is a well-established form of quality assurance and improvement enterprise for undergraduate medical education around the world. The Directory of Organizations that Recognize/Accredit Medical Schools (DORA) has listed 189 accrediting agencies distributed in 129 countries [1]. Despite its pervasiveness, accreditation continues to face criticisms. Accreditation is a summative external evaluation by nature that takes place within a specified schedule (for undergraduate medical education generally between 5 and 10 years in different accrediting agencies). This raises debates between the quality assurance and quality improvement functions of accreditation systems and also between continuous versus episodic forms of reviews in the accreditation process [2]. To tackle these perennial tensions, accreditation authorities has appended a post-accreditation monitoring component to their accreditation process to ensure the continuous quality assurance and improvement of educational programs. Post-accreditation monitoring may include regular reporting, site visits, and other forms of assessment and occurs after an undergraduate medical education program has been accredited in order to monitor the implementation of the recommendations of the professional reviewers given to the accredited program by the standards [3]. Despite the crucial role of post-accreditation monitoring in promoting quality of undergraduate medical education, we have not yet defined such procedures in our undergraduate medical education accreditation system in Iran.

Accreditation relies mainly on the opinion of multiple experts in the form of external visitors and reviewer panels for evaluation and decision making which puts forward other concerns in terms of the consistency and transparency of the processes employed and the usefulness and validity of decisions made. To address these concerns, accreditation, similar to any other evaluations, needs to be reviewed to ensure the quality of its processes and outcomes through meta-evaluation [4]. Meta-evaluation refers to a higher level of evaluation that surpasses individual undergraduate medical education programs and involves assessing the effectiveness and credibility of accrediting bodies themselves. Meta-evaluation can be conducted by external organizations to the accrediting body, a review process is often referred to as ‘meta-accreditation’ or ‘recognition’, or it may be conducted by the accrediting body itself recruiting qualitative or quantitative methodology. Accrediting institutions may apply for meta-accreditation so that their processes for developing standards, conducting external visits and making decisions are reviewed, following the same procedure as accreditation [5]. The World Federation for Medical Education (WFME) is a well-established international body for recognition of accrediting body with growing influences on most of the undergraduate medical education programs. However, its recognition program has been criticized in terms of transparency of procedures, stakeholder participation and the program consequences [6].

The accrediting body for undergraduate medical education in Iran applied for the WFME Recognition Status in 2017 and was awarded the approved recognition status in 2019 after being closely scrutinized by the WFME team [7]. We have also been involved in several internal meta-evaluation activities to ensure the robustness of our accreditation procedures. For instance, Mohassesi et al. examined factors influencing accreditation decisions made within undergraduate medical education accrediting body in Iran to ensure the validity of accreditation decisions [8]. Agabagheri et al. limited the process of monitoring and controlling accreditation activities to standards development and revisions [9] which is only one component of the accreditation systems [10]. There are many other aspects of undergraduate medical education accrediting systems for investigation and there have been calls for scholarship activities in accreditation to provide the evidence base for quality assurance activities [11, 12].

Post-accreditation monitoring, meta-evaluation and meta-accreditation all are essential components of maintaining high-quality undergraduate medical education. However, they supply different functions in the undergraduate medical education accreditation system and have distinct characteristics. While post-accreditation monitoring assesses individual undergraduate medical education programs’ compliance with standards between rounds of accreditations, meta-evaluation and meta-accreditation evaluate accrediting bodies’ adherence to best practices in accreditation processes. Accrediting bodies are involved in meta-evaluation by applying for meta-accreditation or recognition status or by conducting scholarship projects. In this paper, we tried to clarify the confusion surrounding concepts of post-accreditation monitoring, meta-evaluation and meta-accreditation and to provide a better understanding of their roles in the accreditation process. By understanding these distinctions, stakeholders can engage in undergraduate medical education accreditation processes more effectively.

## Data Availability

No datasets were generated or analysed during the current study.
